# Cancer risk before and following organ transplantation

**DOI:** 10.1038/sj.bjc.6602014

**Published:** 2004-07-13

**Authors:** P Maisonneuve, A B Lowenfels

**Affiliations:** 1 Epidemiology Unit, European Institute of Oncology, Via Ripamonti 435, Milan 20141, Italy; 2 Departments of Surgery and of Community and Preventive Medicine, New York Medical College, Valhalla, NY 10595, USA

**Sir**,

Dr Adami *et al* (2003) reported on the risk of cancer following organ transplantation through a nationwide cohort study conducted in Sweden. They reported a persistent, about four-fold increased overall cancer risk and postulated that most of the risks they observed might be attributable to the organ transplantation and subsequent immunosuppressive therapy.

However, we identified an excess risk of cancer in a large cohort of 831 804 nontransplanted end-stage renal disease patients receiving maintenance dialysis in the United States, Europe, Australia and New Zealand (Maisonneuve *et al*, 1999). The distribution of tumour types (excluding lip, nonmelanoma skin cancer and post-transplant lymphomas) that was observed in these nontransplanted patients resembled the pattern generally seen after transplantation. Since, in both studies, the risk of cancer was higher among younger patients, the fact that transplantation is generally offered in younger patients could, in part, explain why slightly higher risks are observed among the transplanted group. Already in 1990, Pecqueux *et al* (1990) studied cancer incidence in patients on chronic dialysis and in renal transplant recipients and concluded that transplantation and consecutive immunosuppression does not appear to constitute an additional cancer risk for the uraemic patient who is faced with either chronic dialysis or renal transplantation.

For the dialysed patients and kidney recipients, we believe that the underlying renal or urinary tract disease, the loss of renal function, or the increased susceptibility to viral carcinogenesis associated with an impaired immune system because of the uraemic state is responsible for most of the increased risk of cancer. The excess of renal parenchymal cancer observed is consistent with causation through acquired renal cystic disease, while the excess of urothelial cancers is consistent with the carcinogenic effects of certain primary renal diseases (Stewart *et al*, 2003). We agree that post-transplant immunosuppressive therapy could be responsible for the development of a large part of the nonmelanoma skin cancers, for cancers of the lip, and for post-transplant lymphomas.

Adami and co-workers also failed to identify a significant excess of cervical cancer, in contrast with a previous report based on similar data (Birkeland *et al*, 1995). Since some cervical cancers could have well been recorded as *uterus unspecified*, it would have been informative to get results for this category.
